# A Year at the Forefront of Proteostasis and Aging

**DOI:** 10.1242/bio.059750

**Published:** 2023-02-16

**Authors:** Maximilian A. Thompson, Evandro A. De-Souza

**Affiliations:** Neurobiology Division, Medical Research Council Laboratory of Molecular Biology, Cambridge, CB2 0QH, UK

**Keywords:** UPR, Cell non-autonomous response, Heat shock response, Longevity, Protein homeostasis

## Abstract

During aging, animals experience a decline in proteostasis activity, including loss of stress-response activation, culminating in the accumulation of misfolded proteins and toxic aggregates, which are causal in the onset of some chronic diseases. Finding genetic and pharmaceutical treatments that can increase organismal proteostasis and lengthen life is an ongoing goal of current research. The regulation of stress responses by cell non-autonomous mechanisms appears to be a potent way to impact organismal healthspan. In this Review, we cover recent findings in the intersection of proteostasis and aging, with a special focus on articles and preprints published between November 2021 and October 2022. A significant number of papers published during this time increased our understanding of how cells communicate with each other during proteotoxic stress. Finally, we also draw attention to emerging datasets that can be explored to generate new hypotheses that explain age-related proteostasis collapse.

## Teaser

**Summary:** This A Year at the Forefront Review summarises key findings and new datasets in the intersection of proteostasis and aging, with a focus on articles and preprints published between November 2021 and October 2022.

## Introduction

Proteostasis is the sum of reactions and signalling pathways related to the synthesis, folding, trafficking, disaggregation, and degradation of proteins (Balch et al., 2008). One of the hallmarks of aging is a decline in proteostasis (López-Otín et al., 2013). Unsurprisingly, defects in all major steps of proteostasis are related to the accumulation of toxic aggregates and misfolded proteins, a key feature of neurodegenerative diseases (Hetz, 2021). Throughout evolution, a range of protein quality-control mechanisms have emerged, some of which are specialised in monitoring the proteome within specific subcellular compartments. Examples are the cytosolic heat-shock response (HSR), the mitochondrial unfolded protein response (UPR^mt^), and the unfolded protein response of the endoplasmic reticulum (UPR^ER^). The mechanisms underlying age-related proteostasis collapse are still not completely understood, but studies using *Caenorhabditis elegans* and mice suggest that it initiates during early adulthood preceding the emergence of age-related diseases (Cabral-Miranda et al., 2022; Taylor and Dillin, 2013). Mechanistically, age-related chromatin changes are implicated in the downregulation of stress-response activity (Labbadia and Morimoto, 2015; Sabath et al., 2020), although recent findings also point to a decrease in the activity of sensors of these pathways (Cabral-Miranda et al., 2022; De-Souza et al., 2022b).

**Fig. 1.**
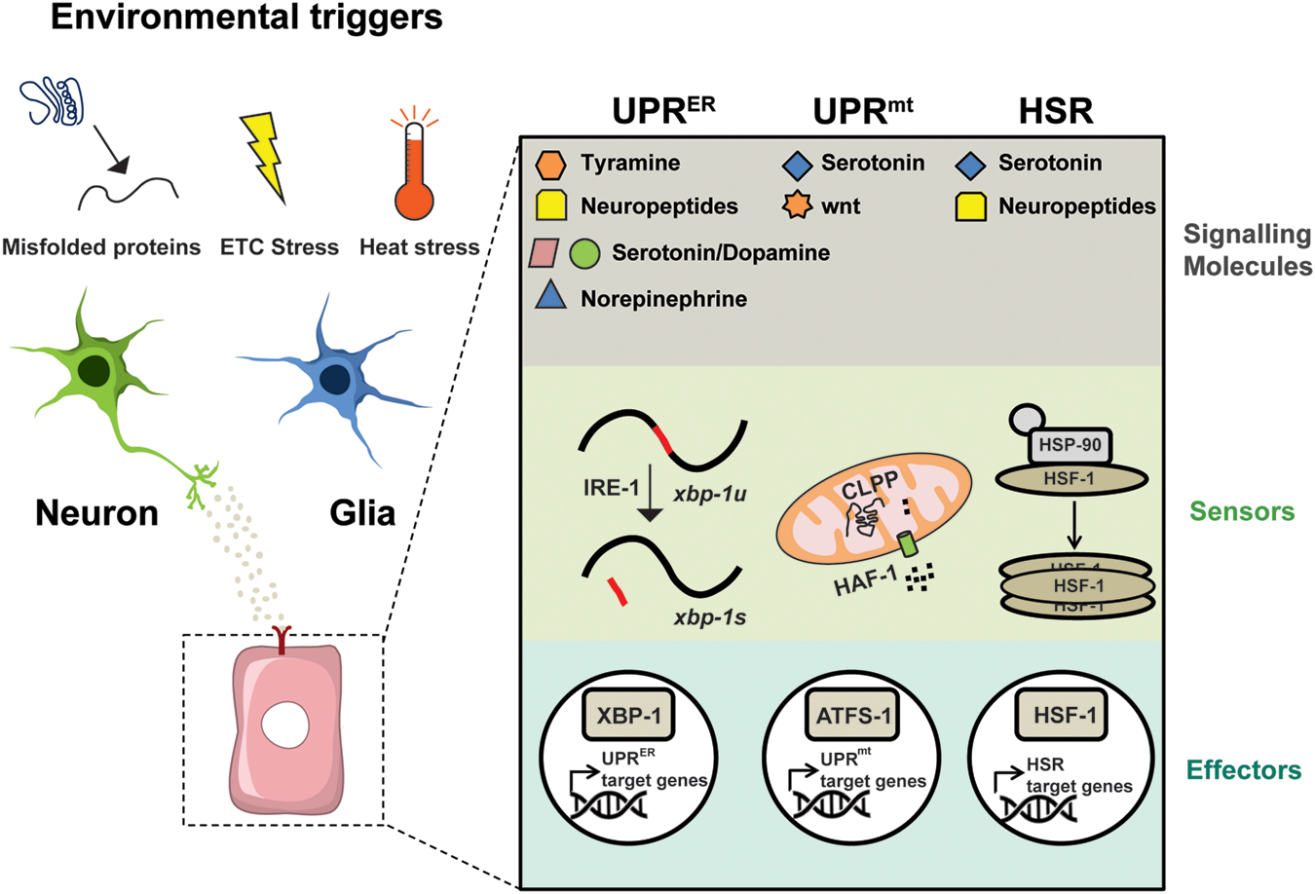
**Cell non-autonomous regulation of proteostasis and longevity by the nervous system.** Both neurons and glia were shown to regulate the HSR, UPR^ER^, and UPR^mt^ in distal tissues in response to genetic interventions or environmental stimulation. This form of signalling depends on the production or secretion of specific neurotransmitters, neuropeptides and other proteins, such as Wnt. Electron Transport Chain (ETC).

For a long time, cellular stress responses that regulate proteostasis (e.g. HSR, UPR) were perceived as signalling pathways that sense disturbances in the local proteome. When misfolded proteins accumulate in organelles, retrograde stress responses are activated to increase the expression of organelle-specific chaperones. However, recent findings, mainly in invertebrates, show that these responses can also be controlled by signals coming from other tissues, especially neurons, in a cell non-autonomous way (Miller et al., 2020) (Fig. 1). Initially, it was found, in *C. elegans*, that upon temperature increase a functional thermosensory neuron was required for the activation of the HSR in other somatic tissues (Prahlad et al., 2008). Neuronal or glial overexpression of XBP-1 (the transcription factor of the UPR^ER^ pathway) also induces the activation of the UPR^ER^ in the distal tissues of the animals (Frakes et al., 2020; Ozbey et al., 2020; Taylor and Dillin, 2013; Williams et al., 2014). Surprisingly, this promotes lifespan extension and protection against toxic protein aggregates (Imanikia et al., 2019; Taylor and Dillin, 2013).

In this Review, we summarise recent advances in the proteostasis and aging fields. We also cover new genetic and pharmacological interventions that have the potential to ameliorate the toxic effects of age-related protein aggregation. Finally, we discuss advances in our understanding of how proteostasis is regulated at a systemic level in metazoans.

## Discoveries

Previous work has established an important relationship between neurons and intestinal cells in the regulation of proteostasis in *C. elegans* (Taylor et al., 2014). Recently, it was shown that neuronal HLH-30/TFEB regulates mitochondrial fragmentation in muscle cells with an impact on organismal thermotolerance (Wong et al., 2022 preprint). Curiously, another study shows that, depending on the nature of the proteotoxic stimulus, worms employ distinct neurons to communicate stress-response activation to somatic tissues (Boocholez et al., 2022). In *C. elegans*, the overexpression of *hsf-1* in the cephalic sheath glia (functional homolog of mammalian astrocytes) was shown in a recent study to increase lifespan, thermotolerance, and pathogen resistance in a mechanism dependent on the intestinal activation of DAF-16 and the HSR (Gildea et al., 2022 preprint). A recent work showed that extracellular vesicles containing ceramides promote UPR^ER^ activation in myotubes in mice (McNally et al., 2022), arguing that some lipid species could regulate proteostasis in distal tissues. Accordingly, new evidence points that sphingolipids can regulate the activity of both the UPR^ER^ and UPR^mt^ (Bieniawski et al., 2022; Yildirim et al., 2022).

Most of the studies investigating cell non-autonomous responses relied on the use of transgenes. Thus, it has not been clear whether this type of response occurs in response to environmental insults. Earlier studies show that the immune system and the HSR pathway in *C. elegans* can both be stimulated by pathogen-associated odour (Ooi and Prahlad, 2017; Prakash et al., 2021). A new study has now observed that olfactory chemosensation of pathogen-derived molecules by chemosensory neurons cause cell non-autonomous activation of the UPR^ER^ in *C. elegans*. This is accompanied by lifespan extension and reduced accumulation of toxic polyglutamine expansions (De-Souza et al., 2022a preprint). In parallel, another group observed that exposure to a specific odour (urine) extends the lifespan of female mice (Garratt et al., 2022 preprint). Identifying strategies for keeping stress responses functional as we age, particularly in the brain, is a promising area of study that may have an impact on age-related disorders. However, this approach is difficult to tune and the blood–brain barrier is a major obstacle in efforts to manipulate neurons. Works showing that neuronal proteostasis can be modulated by sensory inputs (De-Souza et al., 2022a preprint; Ozbey et al., 2020) suggest that, if validated in mammals, targeting chemosensory neurons could be a strategy to bypass the blood–brain barrier to modulate brain proteostasis.

## Technological innovations

Progress in the development of genome editing tools now allows the more comprehensive study of less commonly used model organisms. Advances in knock-in technology using CRISPR were recently obtained in the African turquoise killifish, a vertebrate species that has a short lifespan of just 4-6 months (Nath et al., 2022 preprint). By mutating genes involved in body pigmentation, a transparent killifish reporter line to monitor senescence was created, which could be used to identify new anti-aging interventions (Krug et al., 2022 preprint). Extremophile organisms are characterised by their high resistance to environmental stress, and their study could bring novel insights into the maintenance of proteostasis. Species like tardigrades and naked mole-rats can survive high radiation and prolonged hypoxia, respectively (Little et al., 2021). A series of exciting new studies has used the expression of genes from extremophile species in model organisms. Remarkably, the expression of the hyaluronic acid synthase 2 gene from naked mole-rats in mice promoted lifespan extension (Zhang et al., 2022) and the overexpression of HSPs derived from tardigrades confers desiccation tolerance to bacteria (Hibshman et al., 2023). Recently, a CRISPR protocol was developed for genome editing in tardigrades (Kumagai et al., 2022), which will allow investigators to test the function of specific proteins in their extremophile phenotype.

The last year has also seen important progress in the development of aging biomarkers. Historically, epigenetic clocks were developed by using the DNA methylation patterns in specific genomic regions as a way to measure biological aging (Horvath, 2013). This year, a consortium generated a functional epigenetic clock applicable to different mammalian species (Lu et al., 2021 preprint). The authors of this study argue that the fact that they are able to create an epigenetic clock which works across species suggests the existence of an underlying process driving aging, conserved across diverse mammalian species. Another study, in humans, generated a physiology clock refining from 120 to only 12 physiological parameters (e.g. systolic blood pressure, IGF-1 levels, etc.) that when tracked collectively serve as a reliable predictor of biological age (Libert et al., 2022 preprint). The continued development of reliable biomarkers will be essential for clinical trials evaluating interventions that can lengthen human healthspan in the coming years.

## New resources

Single-cell RNA sequencing of *C. elegans* during aging was independently performed by two groups (Roux et al., 2022 preprint; Wang et al., 2022). Both studies found altered levels of the UPR^ER^-related transcriptional factor XBP-1, indicating that ER proteostasis is differentially regulated during aging. Tissue-specific proteome profiling was also performed in killifish and mice, and curiously both studies found alterations in the levels of some ribosomal subunits (Chen et al., 2022a preprint; Keele et al., 2022 preprint). In addition, a significant number of databases related to aging and proteostasis were published in the last year (Table 1).

**Table 1.**
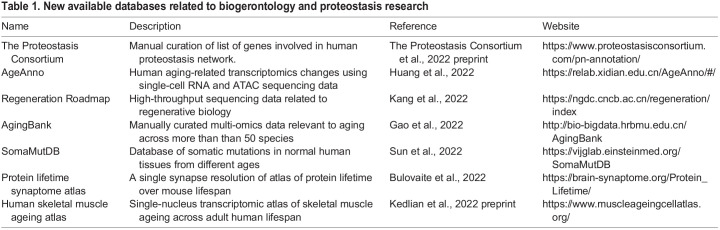
New available databases related to biogerontology and proteostasis research

## New hypotheses

A fundamental question in biogerontology is why animals lose the ability to maintain proteostasis with aging. A new study observed that an age-related increase in ribosome pausing occurs driven by a reduced activity of the ribosome quality control (RQC) pathway (Stein et al., 2022). Another provocative study, in mice, argued that error-prone translation caused by ribosomal ambiguity mutations induces phenotypes that more closely match the progression of the Alzheimer's Disease than Aβ amyloid overexpression models do (Brilkova et al., 2022). Their findings suggest that the accumulation of random mutations in DNA over time may induce increased protein misfolding, sequestering away key components of the proteostasis maintenance machinery, such as chaperones, ultimately causing a collapse in proteostasis.

The use of model organisms for aging research allows the direct study of longevity over reasonable timescales that would not be practicable using humans. The main model organisms used for lifespan studies are the nematode *C. elegans*, the fruit fly *Drosophila melanogaster*, and the mouse *Mus musculus*. While these organisms vary in complexity, the rationale for choosing them for lifespan studies is that these animals have fast development and are short-lived. Recent work, however, suggests that *C. elegans* is a semelparous species, undergoing reproductive death (Kern and Gems, 2022). This might have significant implications since it increases the chance that interventions that extend worm's lifespan will not translate to higher organisms, and underscores the importance of understanding the evolutionary logic at work in the models used to study aging. The authors of this study further argue that semelparity and iteoparity likely exist on a continuum rather than as fully distinct categories (Kern and Gems, 2022; Kern et al., 2020 preprint). Many researchers view mouse lifespans as the gold standard of a bona fide pro-longevity intervention. While mice live longer than *C. elegans*, *M. musculus* was also chosen as a model organism in part due to its short generation time and large litters (Phifer-Rixey and Nachman, 2015), suggesting that (although there is no evidence of semelparity in mice) even the mouse employs a very different life strategy from humans.

A popular assumption in the biogerontology field is that there is a single underlying mechanism which comprises ‘aging’ that if addressed directly could delay or even reverse aging itself. Building on earlier theories of entropic aging, a recent analysis of DNA methylation data and longitudinal medical records identifies thermodynamic biological age (tBA) (Tarkhov et al., 2022 preprint) as a good readout of an organism's aging state, positioning increasing entropy as the major underlying process that drives aging. This suggests that some approaches that seek to reverse aging without replacing significant parts of the organism may be limited in their success, and therefore supports the hypothesis that interventions to target aging will be more effective if they slow the rate of aging itself, rather than seeking to reverse aspects of aging. Similarly, a recent review advances the hypothesis that diverse types of molecular damage (including many that impact proteostasis) cumulatively underlie aging (Gladyshev et al., 2021).alz

## Future prospects

One exciting prospect on the horizon for aging research is the arrival of data on aging interventions from several large-scale studies. Companion dogs, in contrast to laboratory animals, are genetically diverse and – much like their human owners – live in diverse environments. Several efforts to study the effects of various pro-longevity interventions in dogs are underway, notably the dog aging project which is studying the effect of rapamycin in dogs (Urfer et al., 2017) and studies underway in the company Loyal (Chen et al., 2022b preprint). Thus, the arrival of data on the effectiveness of pro-longevity interventions in pets has the potential to provide the strongest evidence to date for the feasibility of pharmacological human lifespan extension. A human clinical trial testing the FDA-approved compound metformin for lifespan extension is well underway and the arrival of this data will represent the first major trial of a drug for lifespan extension in humans, regardless of the outcome (Barzilai et al., 2016). Finally, in the field of Alzheimer's Disease, lecanemab caused a small but significant positive result in a phase IIb clinical trial in humans suffering from Alzheimer's Disease, and it is expected that results from the phase III study will be available soon (Swanson et al., 2021). Lecanemab is a monoclonal antibody which targets soluble Aβ, and the development of a bona fide treatment for Alzheimer's Disease, if ultimately successful represents an important milestone in neurodegenerative disease research.

Therefore, together with the emergence of novel aging biomarkers, we expect that ongoing clinical trials will be able to test hypotheses and interventions generated in the last decades with the potential impact to extend the human healthspan. Together with the development of novel datasets and hypothesis, we expect an increase in our understanding of the relationship between proteostasis and aging in the next few years.
